# Enhancing undergraduate research talents: the role of tutors in dental basic research education

**DOI:** 10.3389/fmed.2023.1323183

**Published:** 2024-01-08

**Authors:** Xiaoyu Miao, Xuanyu Chen, Jiayi Li, Zhe Wu, Lvhua Guo, Siqi Luo, Tao Luo, Xuesong Yang

**Affiliations:** ^1^School of Stomatology, Guangzhou Medical University, Guangzhou, China; ^2^Department of Prosthodontics, Guangzhou Key Laboratory of Basic and Applied Research of Oral Regenerative Medicine, Guangdong Engineering Research Center of Oral Restoration and Reconstruction, Affiliated Stomatology Hospital of Guangzhou Medical University, Guangzhou, China; ^3^Clinical Research Center, Clifford Hospital, Guangzhou, China; ^4^Division of Histology and Embryology, Medical College, Jinan University, Guangzhou, China

**Keywords:** mentorship, medical education, feedback, academic performance, scientific research

## Abstract

**Purpose:**

This study endeavors to investigate ways to optimize the role of teachers in undergraduate dental basic research education (UDBRE) with the aim of nurturing the research potential of undergraduate students.

**Methods:**

We conducted a cross-sectional study among medical undergraduates enrolled at the School of Stomatology, Guangzhou Medical University. Descriptive statistics were employed to comprehensively analyze UDBRE’s fundamental aspects. Kendall rank correlation analysis was performed to evaluate the relationship between the quality of feedback provided by tutors to undergraduates and the students’ scientific research abilities. Additionally, multivariate logistic regression analysis was employed to uncover the factors influencing the effectiveness of UDBRE.

**Results:**

A total of 168 medical students were surveyed with a valid response rate of 93.85%. The effectiveness of UDBRE was demonstrated by undergraduates’ self-rated research abilities, active participation in scientific research projects, and a certain amount of academic outputs. Significant and positive correlations (𝓣_b_*>* 0.5*, p* < 0.001) were identified between the tutor-undergraduate feedback quality and students’ self-rated scores for scientific research abilities. These abilities included developing scientific questions, designing research projects, retrieving and reading literature, academic writing, experiment operation, and analyzing and evaluating experimental results. Positive effects on students’ academic performance (*p* < 0.05) were observed when higher-quality feedback, an authoritative tutoring style and tutors with middle-career experience were present.

**Conclusion:**

This study underscores the pivotal role of UDBRE in fostering the scientific research aptitude of medical undergraduates. It emphasizes the constructive influence of tutor-undergraduate feedback, authoritative teaching styles, providing valuable insights for establishing an effective mentorship framework.

## Introduction

In an era marked by an explosion of information, it is imperative that medical students acquire the ability to critically assess scientific papers (1). To achieve this goal, it is paramount for them to comprehend the origins of information and systematically evaluate pertinent studies. The idea of embedding research into the dental curriculum dates back to 1926 (2). Engaging in research during collegiate education can foster active learning and critical thinking, enabling students to identify scientific challenges in routine clinical practice. Opportunities for research projects have been extended to dental graduates to champion dental advancements in European and American countries, as well as China (3–5). However, a significant proportion of dental graduates gravitate towards private practice rather than a research career (6). To bolster student engagement in scientific research, various strategies have been proposed during undergraduate studies (7). The concept of “Undergraduate Dental Basic Research Education (UDBRE)” has been globally embraced in curricula, with UDBRE designed to cultivate research interest and inspire creativity in students’ future careers (8, 9).

Typically UDBRE refers to a pedagogical strategy that integrates theoretical coursework with active participation in mentor-guided student research projects and hands-on training in experimental techniques (9). A standard paradigm has been outlined by Eryi Lu (10). Under the premise of two-way selection between tutors and students, highly professional and morally excellent teachers are appointed as tutors for undergraduate students, providing individual guidance on their academic studies, and grant drafting. Undergraduate student tutor and student communicate regularly and student report prepared work on literature reading, reading and essays. UDBRE plays a vital role in guiding students through scientific projects, and providing invaluable assistance, suggestions, and guidance for students’ research ideas and designs (9). This approach not only stimulates intellectual curiosity and latent capabilities but also fosters the early development of research acumen and skills (11, 12). The primary objective of the present study is to assess the effectiveness of UDBRE using both objective measures of academic performance and a questionnaire designed to evaluate academic capabilities.

The paradigm of undergraduate training with a focus on academic research has ushered in a significant shift from a teacher-centered approach to a more student-centered educational model (13, 14). This transition towards a student-centered approach may sometimes result in minimal intervention, potentially leading to a neglectful tutor style. Within this context, scholars have advocated for the incorporation of dialogic approaches, characterized by open and interactive dialogues between tutors and students, followed by authoritative interventions within the realm of scientific training (15). However, the influence of tutor style on students’ research abilities remains an area yet to be fully explored.

Furthermore, scientific abilities encompass a broad spectrum of proficiencies, ranging from proficient literature comprehension and refined academic writing to the skillful execution of experiments and more. These demands pose significant challenges for students, especially as they must balance these requirements with their dental studies (16). This challenge becomes particularly pronounced for Chinese students who often grapple with materials predominantly presented in English (17). Therefore, it is imperative to approach student involvement in research with a heightened emphasis on promoting self-motivated engagement. The attitudes and teaching methodologies of instructors are closely linked to shaping students’ levels of motivation (18). Negative attitudes demonstrated by educators have been found to correlate with reduced student motivation (19). Rather than adopting a narrow focus solely on evaluating the accuracy of students’ work or appraising their aptitudes, an effective approach hinges on the provision of constructive feedback. This approach entails imparting informative feedback that not only affirms students’ accomplishments but also highlights areas where improvements can be made. This practice offers the dual benefit of bolstering students’ self-assurance and fostering the refinement of their skill sets (20). The feedback-driven approach also contributes to the cultivation of a robust sense of self-assuredness among students while simultaneously enhancing their competencies. Therefore, the second aim of this study is to explore the relationship between feedback given by students and teachers.

In order to provide effective guidance in UDBRE and meet the mentorship needs of students participating in UDBRE, it is crucial to identify more successful forms of mentoring and cost-effective improvements, especially within large research universities. To address this, we conducted a cross-sectional survey aimed at confirming the effectiveness of UDBRE and further investigating a series of indicators, including feedback from both tutors and students, tutoring styles, and career experience. The goal is to determine whether these indicators can signify a successful mentoring relationship, thereby contributing to the reform and development of UDBRE in colleges and universities.

## Methods

### Description of UDBRE

UDBRE have been piloted since 2016 to now in School of Stomatology, Guangzhou Medical University. The primary objective of this dental undergraduate research program is to promote and facilitate undergraduate students’ engagement in drafting and applying for research grants, conducting research, and publishing peer-reviewed papers. The initial step involved soliciting faculty members from the School of Stomatology Guangzhou Medical University to volunteer as mentors for students. Subsequently, a list of willing mentors was provided to students, initiating a two-way selection process between tutors and students. To equip students with the necessary skills and knowledge, a dedicated course on innovation experiments was integrated into the curriculum during the third grade. This course covered fundamental concepts essential for fostering basic research and innovation skills, including topics such as research subject exploration, research methodology, literature review techniques, experimental design and execution, data analysis, and academic writing. As part of this course, students were tasked with preparing a research grant proposal and a research paper. Furthermore, they were strongly encouraged to actively apply for research grants and submit their papers to peer-reviewed journals, all under the expert guidance of their assigned tutors. Each research tutor is obligated to provide continuous mentorship to undergraduate students, ensuring that academic achievements are submitted and that students have the opportunity to present their research findings effectively.

### Participants and ethics approval

From 2016 to the present, a total of 311 undergraduate students enrolled in the School of Stomatology at Guangzhou Medical University actively participated in the Undergraduate Dental Basic Research Education (UDBRE) program. To comprehensively assess the impact of this training initiative, we analyzed their achievements, including published papers, approved research grants, and awards. Additionally, we conducted a cross-sectional survey among 179 undergraduate students who were in their third year or higher, utilizing a questionnaire to gain insights into their experiences and perceptions related to UDBRE. 168 (93.85%) of students responded with complete answers. Prior to their participation in the study, each student was provided with a comprehensive information sheet outlining the study’s purpose, objectives, the nature of their involvement, the expected duration of participation, potential risks and benefits, and details regarding data confidentiality. It was made explicit that the information provided would not be shared with their teachers, nor used to assess their abilities to prevent potential bias. Each student was required to read the information sheet thoroughly to ensure a clear understanding of their involvement. Subsequently, they provided written informed consent indicating their voluntary agreement to participate in the study. The proposal for this study was approved by the institutional ethics committee of Guangzhou Medical University (Ref No. LCYJ2023019).

### Questionnaire design

The questionnaire was designed to consist of 29 items and contain three sections in Chinese, including basic information, the academic outcome and self-rated scientific research competency of undergraduates.

In section one, characteristic data of the participating undergraduates was collected including gender of undergraduates, grade, the tutoring style, the tutor’s career experience, the average tutoring time. The definition of 4 types of tutoring style is based on existing literature and presented in the form of a multiple-choice question, allowing students to select the most suitable type based on their actual experiences (21). The authoritative tutor is characterized by a high level of demandingness and expectations, coupled with an active involvement in student’s learning. The authoritarian style entails high levels of demandingness but low levels of involvement, whereas the permissive style displays the inverse with high involvement but low demandingness. The average tutoring time included mentorship conducted by online meetings, face-to-face interactions, and other means.

The second section targeted the correlation relationship between the tutor-undergraduate feedback quality and undergraduates’ scientific research abilities. Feedback quality between tutors and undergraduates was evaluated using a 5-point Likert scale ranging from 1 (strongly disagree) through 3 (neutral) to 5 (strongly agree). Based on existing literature (22), this self-made scale comprised 6 items grouped under two dimensions, named “feedback quality of tutors” and “feedback quality of undergraduates.” A 5-point Likert scale was administered to the interviewees to learn their own perception of the development of their academic and research skills including scientific questions developing, scientific research projects design, literature retrieving and reading, academic writing, experimental operation and experimental results analyzing and evaluating. And the scale ranged from 1 (strongly disagree) to 5 (strongly agree).

As the goal of UDBRE in Guangzhou Medical University is to instruct students to publish academic papers and applying for funded research projects of variety of kinds, the last section reflected the academic outcome of participated undergraduates including completed academic paper drafts, published papers as first or co-first authors, funded research projects and awards in competition. Completed article draft is a shorter-term indicator of academic performance. In the evaluation of published academic achievements, the students provided the number of papers as first authors or co-first authors, which had been published. As for funded research projects, we counted the number of the students who presided or participated in scientific research projects mainly including the National College Students Innovative Training Program and College Students Innovative Training Program of Guangdong Province. Competitions, such as the China College Students’ “Internet plus” Innovation and Entrepreneurship Competition and the “Challenge Cup” College Students’ Extracurricular Academic Science and Technology Works Competition, were included for a descriptive analysis of UDBRE. The number of completed academic paper drafts, published research articles, and funded research projects were taken as dependent variables of multiple logistic regression analysis. Each item was graded as 1, none; 2, one; and 3, two or more. At last, an open-ended question was used to gather detailed thoughts from students about their UDBRE participation.

### Questionnaire reliability and validity analysis

The complete questionnaire was meticulously reviewed for relevance, comprehensiveness, as well as face and content validity prior to the commencement of data collection. Pilot testing was also done among 30 students to ensure clarity of the content of the questionnaire. The internal consistency reliability test was performed to ensure the overall reliability of the questionnaire. Cronbach’s α coefficients for the tutor-undergraduate feedback scale and scientific research ability scale were 0.892 and 0.967, respectively, which exceeded the commonly used threshold of 0.7 for good reliability. Exploratory factor analysis (EFA) was performed to specify the structure and underlying dimensions of the scale. The Kaiser-Meyer-Olkin (KMO) measure of sampling adequacy and Bartlett’s sphericity test were performed to assess the eligibility to EFA. The EFA was conducted through the principal component analysis to extract the main factors may contribute to the variance in the overall samples from 6 items based on the eigenvalue>1. Varimax Kaiser normalization was used to rotate the factor load matrix. The KMO coefficient of scientific research ability scale was 0.928 and Bartlett’s sphericity test was statistically significant (χ^2^ = 1506.072, *p* < 0.001), which indicated a well-constructed structure.

### Statistical analysis

Descriptive statistics for this data was presented as number, constituent ratio and mean ± standard deviation (SD). The average points of the Likert scores for each item/factor were presented as the mean ± SD. Multivariate ordinal logistic regressions were used to find the associated factors for completed academic paper drafts, published papers and funded research projects of undergraduates. The test of parallel lines is used in the context of analysis of variance (ANOVA) or analysis of covariance (ANCOVA) to examine if the slopes of multiple regression lines are parallel. The Kendall rank correlation analysis was applied to evaluate correlation between the tutor-undergraduate feedback quality and the undergraduates’ self-perceived scientific research abilities. Graphical analysis of descriptive data was conducted using GraphPad Prism 9 software version 9.5.0 (La Jolla, CA, Unites States). Data were analyzed using SPSS version 25.0 (IBM, Unites States) and *p* < 0.05 was considered a statistically significant difference.

## Result

### Basic information of participants

The basic information of participants is shown in Table 1. 92 (54.76%) participants were male and 76 (45.24%) were female. A total of 63 (37.50%) students were from the third grade, 49 (29.17%) were from the fourth grade and 56 (33.33%) were from the fifth grade. As for the average weekly tutoring time, over half of students (N = 87, 51.79%) were received mentorship of less than 15 min. 71 (42.26%) students had scientific projects approved, but the number decreased to 61 (36.31%) and 43 (25.60%) when it comes to students with academic paper drafts for publication and published articles as first or co-first authors, respectively.

**Table 1:** Basic information of participants.

Characteristic	Number (*N*)	Constituent ratio (%)
**Gender of undergraduates**		
Male	92	54.76
Female	76	45.24
**Grade**		
Third grade	63	37.50
Fourth grade	49	29.17
Fifth grade	56	33.33
**Career experience**		
Late career (>10 years)	40	23.81
Middle career (6–10 years)	62	36.90
Early career (<6 years)	66	39.29
**Tutoring style**		
Authoritative	61	36.31
Authoritarian	19	11.31
Permissive	46	27.38
Neglectful	42	25.00
**Average weekly tutoring time**		
≥15 min	81	48.21
<15 min	87	51.79
**With funded scientific projects**		
Yes	71	42.26
No	97	57.74
**With completed academic paper drafts**		
Yes	61	36.31
No	107	63.69
**With published articles**		
Yes	43	25.60
No	125	74.40

### Descriptive analysis of the academic outcome of undergraduates since UBDRE implementation

**Figure 1:**
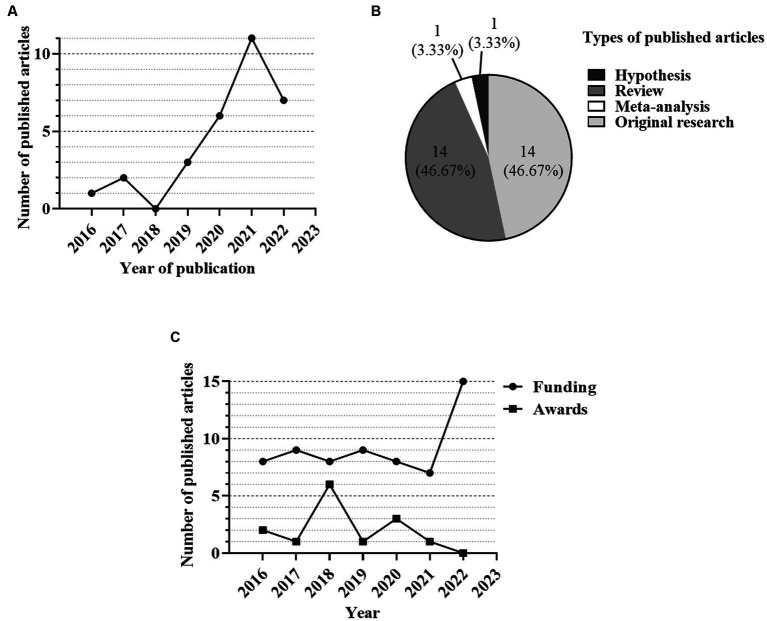
Academic outcome of undergraduates in UDBRE from 2016 to 2022. **(A)** The number of published articles by undergraduates as first authors or co-authors over the years. **(B)** Types of published articles. **(C)** The number of funded research projects and awards in competition obtained by undergraduates.

From 2016 to 2022, undergraduates participating in UDBRE achieved notable academic milestones, which encompassed publications in peer-reviewed journals, successful grant-funded research projects, and recognition through awards in innovation and entrepreneurship competitions. The findings underscored a consistent upward trajectory in the number of research papers authored by undergraduates, whether as independent first authors or co-first authors. This journey commenced with just one paper in 2016 and culminated in an impressive 11 papers in 2021, as illustrated in Figure 1A. Consequently, by 2022, the total count of research papers authored by UDBRE students had reached a commendable tally of 30. It is noteworthy that the majority of these publications were comprised of reviews and original research articles, constituting 14 (46.67%) each of the total count, as depicted in Figure 1B.

Furthermore, we documented the involvement of undergraduate students in funded research projects and innovation and entrepreneurship competitions. Over the years, the number of research projects funded for undergraduates maintained a relatively high and stable level (Figure 1C). And it achieved a new breakthrough in 2022, reaching a total of 15 projects. This significant progress could be largely attributed to the students’ participation in the introduced enhancement program. In total, undergraduates had actively participated in 64 research projects from 2016 to 2022. Regarding the number of awards obtained by undergraduates in innovation and entrepreneurship competitions, although this figure experienced a decline in 2021 and 2022, students collectively won 14 awards.

### Exploratory factor analysis of the tutor-undergraduate scale

The results of the EFA indicated that the scale demonstrated good structural validity. A total of six items were subjected to exploratory factor analysis (Table 2). The KMO coefficient was 0.843, approaching 1, which indicated that the data were suitable for EFA. Additionally, Bartlett’s sphericity test was statistically significant (χ^2^ = 649.21, *p* < 0.001) allowing the EFA to be performed (23). Two common factors were extracted using principal component analysis, which accounted for a cumulative variance explained rate of 84.30%. This indicated that these factors could explain 84.30% of all items and effectively capture most of the information conveyed by the indicators. Furthermore, all factor loading values exceeded 0.7, meeting the criteria for excellent factor loading (24). Therefore, the tutor-undergraduate feedback scale demonstrated good structural validity. The two dimensions identified through factor analysis were labeled as “Feedback quality of tutors” and “Feedback quality of undergraduates,” representing the reciprocal feedback between tutors and undergraduates. The average scores for the feedback quality of tutors and undergraduates were 2.82 and 2.87, respectively.

**Table 2:** Exploratory factor analysis factor component matrix of tutor-undergraduate feedback scale.

Item	Factor loading	
	Factor 1: feedback quality of tutors	Factor 2: feedback quality of undergraduates
1. The tutor responds positively to your feedback	0.873	
2. The tutor can understand your feedback correctly	0.872	
3. The tutor’s feedback can help solve your problem	0.853	
4. You often proactively give feedback to your tutor		0.796
5. You can provide feedback on your own issues accurately		0.860
6. You can provide continuous feedback to your tutor during the problem-solving process		0.893
*n*	3	3
Cronbach’s α coefficient	0.879	0.893
Cumulative variance percent (%)	43.956	40.352
Score (mean ± SD)	2.82 ± 1.65	2.87 ± 1.47

Score: mean scores of each item of the factor; *n*: number of the related items of each factor; SD: standard deviation.

### Correlation analysis between the tutor-undergraduate feedback quality and scientific research ability

The evaluation of students’ research abilities involved the use of a 5-point Likert scale. Kendall rank correlation analysis was then conducted to examine the relationship between the tutor-undergraduate feedback quality and students’ research abilities (Table 3). The Kendall Tau-b coefficient (𝓣_b_) was used to calculate correlation scores, ranging from 0 (indicating no correlation) to 1 (a complete correlation). The correlation coefficient between tutor-undergraduate feedback quality and scientific research ability was revealed to range from 0.543 to 0.660, which indicated that the survey items exhibited close correlations. Of note, the tutor-undergraduate feedback quality was most positively correlated with the ability of designing scientific projects (𝓣_b_ = 0.660 and 0.652 respectively), while the experimental operation skill showed weakest correlation (𝓣_b_ = 0.522 and 0.543 respectively).

**Table 3:** Correlation between tutor-undergraduate feedback quality and undergraduates’ scientific research abilities.

	Students’ feedback quality	Tutors’ feedback quality	Scientific questions developing	Scientific research projects design	Literature retrieving and reading	Academic writing	Experimental operation
Tutors’ feedback quality	0.689**						
Scientific questions developing	0.615**	0.598**					
Scientific research projects design	0.652**	0.660**	0.788**				
Literature retrieving and reading	0.602**	0.577**	0.719**	0.780**			
Academic writing	0.646**	0.636**	0.755**	0.829**	0.845**		
Experimental operation	0.543**	0.522**	0.587**	0.649**	0.570**	0.597**	
Experimental results analyzing and evaluating	0.591**	0.573**	0.659**	0.729**	0.674**	0.712**	0.769**

Correlations were expressed as Kendall Tau-b coefficients (𝓣b). **p < 0.01.

### Multivariate logistic regression analysis of influential factors of academic outcome

To investigate the influential factors of students’ academic performance including the number of completed academic paper drafts, published articles, funded research projects, and interviewees’ basic characteristics were taken as independent variables. The two dimensions of “feedback quality of tutors” and “feedback quality of undergraduates” were taken as continuous independent variables of multivariate logistic regression.

The result of the ordinal logistic regression analysis showed that the feedback quality of tutors and undergraduates, the tutoring style, and the career experience were significantly related to the student’s completed academic paper draft (Table 4). The result of parallel line test was *p* > 0.05, rejecting the parallel lines assumption, which indicated that ordinal logistic model could be used. The odds ratio (OR) is used to quantify the strength and direction of the association between explaining variables and the specific dependent variable. It compares the odds of an event occurring in a subcategory group to the odds of the same event occurring in another subcategory group (reference category). When the OR is greater than 1, it indicates that the positive outcome is more likely to occur in the subcategory group compared to the reference group. In other words, there is a positive association between the subcategory group being studied and the dependent variable. The OR for explaining variables was adjusted for grade of undergraduates, which was considered a confounding factor. The result showed that high-quality feedback from tutors [OR = 1.898, 95% confidence interval (CI) 0.050–1.232, *p* = 0.033] and students (OR = 1.963, 95% CI 0.005–1.343, *p* = 0.048) both had a significant positive predictive effect on students’ academic paper drafts. The result also indicated that authoritative tutors could positively influence academic achievements, with a 6.938 times higher likelihood of completing drafts compared to neglectful tutors (OR = 6.938, 95% CI 0.368–3.506, *p* = 0.016). Moreover, students with mid-career tutors (OR = 5.476, 95% CI 0.459–2.942, *p* = 0.007) were 5.476 times more likely to have better performance in the number of academic paper drafts compared to those with early-career tutors.

**Table 4:** Multivariate logistic regression analysis of influential factors of completed academic draft for publication.

Explaining variable	B	S.E	Wald	*p*-value	OR	95% CI	
						Lower limit	Upper limit
**Feedback quality of tutors**	0.641	0.301	4.521	0.033*	1.898	0.050	1.232
**Feedback quality of undergraduates**	0.674	0.341	3.901	0.048*	1.963	0.005	1.343
**Tutoring style**							
Authoritative style = 1	1.937	0.800	5.858	0.016*	6.938	0.368	3.506
Authoritarian style = 2	1.148	1.149	0.999	0.317	3.153	−1.103	3.400
Permissive style = 3	−0.712	0.903	0.622	0.430	0.491	−2.481	1.058
Neglectful style = 4	Reference category						
**Career experience**							
Late career = 1	−0.350	0.810	0.186	0.666	0.705	−1.937	1.237
Middle career = 2	1.700	0.634	7.203	0.007**	5.476	0.459	2.942
Early career = 3	Reference category						
**Dependent variable**							
**Completed research draft**							
None =1	−2.651	1.271	4.349				
One = 2	7.002	1.427	24.08				
Two or more = 3							

Test of parallel lines: *p* = 0.986, pseudo *R*^2^ = 0.598, Variance Inflation Factor (VIF) = 3.776. Results were adjusted for grade. Values in bold showed the statistically significant difference. *p < 0.05, **p < 0.01. S.E: standard error; OR: odds ratio; CI: confidence interval.

With the analysis of the academic publications as first or co-first authors, it was observed that several factors significantly influenced the outcome, including the feedback quality of tutors, the tutoring style and the tutor’s career experience (Table 5). The feedback quality of tutors emerged as a positive impact factor (OR = 3.556, 95% CI 0.342–2.196, *p* = 0.007). That is, every one point increased in the feedback quality score of tutors was associated with a 3.556 times higher likelihood of publishing more papers. Furthermore, students mentored by authoritative tutors demonstrated a higher tendency in publications compared to those mentored by neglectful tutors, with an OR value of 12.829 (OR = 12.829, 95% CI 0.107–4.996, *p* = 0.041). Additionally, the OR of middle-career tutors compared to early-career tutors was 10.371 (OR = 10.371, 95% CI 0.749–3.929, *p* = 0.004), indicating that undergraduates mentored by middle-career tutors were ten times more likely to publish academic achievements than those mentored by early-career tutors.

**Table 5:** Multivariate logistic regression analysis of influential factors of published academic achievement.

Explaining variable	B	S.E	Wald	*p*-value	OR	95% CI	
						Lower limit	Upper limit
**Feedback quality of tutors**	1.269	0.473	7.196	0.007**	3.556	0.342	2.196
**Feedback quality of undergraduates**	−0.389	0.436	0.797	0.372	0.678	−1.243	0.465
**Tutor’s style**							
Authoritative style = 1	2.552	1.247	4.185	0.041*	12.829	0.107	4.996
Authoritarian style = 2	1.822	1.663	1.200	0.273	6.185	−1.438	5.082
Permissive style = 3	−1.160	1.668	0.483	0.487	0.314	−4.429	2.110
Neglectful style = 4	Reference category						
**Career experience**							
Late career = 1	0.701	1.177	0.355	0.552	2.015	−1.606	3.008
Middle career = 2	2.339	0.811	8.317	0.004**	10.371	0.749	3.929
Early career = 3	Reference category						
**Dependent variable**							
**Published academic achievement**							
None =1	6.895	2.020	11.657				
One = 2	10.582	2.194	23.266				
Two or more = 3							

Test of parallel lines: *p* = 0.995, pseudo *R*^2^ = 0.520, VIF = 3.776. Results were adjusted for grade. Values in bold showed the statistically significant difference. *p < 0.05, **p < 0.01. S.E: standard error; OR: odds ratio; CI: confidence interval.

As for the funded research projects, it was found to be influenced by the feedback quality between tutors and undergraduates and the tutor’s career experience according to the findings of ordinal logistic regression (Table 6). Improvements in the quality of tutor-student feedback were found to effectively increase the number of projects, respectively (OR = 2.917, 95% CI 0.528–1.613, *P*<0.001 and OR = 1.902, 95% CI 0.018–1.268, *p* = 0.044). Taking the tutor with neglectful style for reference, the authoritative tutors were 5.244 times more likely to improve students’ performance in funded research projects by at least one level (OR = 5.244, 95% CI 0.300–3.014, *p* = 0.017).

**Table 6:** Multivariate logistic regression analysis of influential factors of funded research projects.

Explaining variable	B	S.E	Wald	*p*-value	OR	95% CI	
						Lower limit	Upper limit
**Feedback quality of tutors**	1.070	0.277	14.939	<0.001***	2.917	0.528	1.613
**Feedback quality of undergraduates**	0.643	0.319	4.068	0.044*	1.902	0.018	1.268
**Tutor’s style**							
Authoritative style = 1	1.657	0.692	5.729	0.017*	5.244	0.300	3.014
Authoritarian style = 2	0.219	0.967	0.051	0.821	1.245	−1.677	2.115
Permissive style = 3	−0.698	0.772	0.816	0.366	0.498	−2.211	0.816
Neglectful style = 4	Reference Category						
**Career experience**							
Late career = 1	−0.516	0.723	0.509	0.475	0.597	−1.932	0.901
Middle career = 2	−0.344	0.638	0.291	0.590	0.709	−1.595	0.907
Early career = 3	Reference Category						
**Dependent variable**							
**Published academic achievement**							
None =1	5.890	1.216	23.469				
One = 2	7.401	1.298	32.516				
Two or more = 3							

Test of parallel lines: *p* = 0.683, pseudo *R*^2^ = 0.625, VIF = 3.776. Results were adjusted for grade. Values in bold showed the statistically significant difference. *p < 0.05, ***p < 0.001. S.E: standard error; OR: odds ratio; CI: confidence interval.

## Discussion

UDBRE represents an innovative approach to enhancing research experiences within undergraduate education, and this study reaffirms its effectiveness. What sets this initiative apart from many others is the requirement for students to complete a compulsory course, thereby ensuring that research experiences are accessible to a broader spectrum of undergraduates, regardless of their prior knowledge of research methodologies. Recognizing the potential constraints related to time and resources that may impede the scalability of UDBRE, we explore strategies for improving guidance, with a primary focus on the pivotal role of feedback, tutoring style, and the experience of mentors.

In the student-centered model of UDBRE, the primary role of mentors is to facilitate the learning process, encourage collaboration, and provide formative feedback rather than impart factual knowledge (25). Teaching with feedback appears to be a promising solution based on the challenges posed by the lack of mentorship, where only 48.21% tutors provided more than 15-min mentorship per week, and each tutor was responsible for multiple grades of students. High-quality feedback between tutors and students is considered to be the students’ motivation to continuous implementation of research project, and to promote students’ progress in scientific research (26). When learners have a clear understanding of what they are being assessed on and regularly receive explicit feedback from others regarding their progress and abilities, they are more likely to provide more effective responses (27). Our study found that high-quality feedback of tutors and students had a significant positive effect on their own academic achievements, emphasizing the importance of appropriate feedback. Of note, compared with other abilities, the correlation between feedback quality and the improvement in experimental operation was weaker. This was possibly due to the outbreak of COVID-19, which resulted in students’ inaccessibility to laboratories, and therefore they had no opportunities for experimental training (28). While the training of other skills could be carried out online without disruption. According to the open-ended responses, students hoped to obtain more opportunities to experience and learn more diversified experiments which required us to explore a more reasonable laboratory training system.

Appropriate teaching styles facilitate the establishment of an effective teaching atmosphere, improve the quality of feedback between tutors and undergraduate students, and finally promote academic progress (29). Previous studies have already demonstrated that the authoritative style is the most beneficial for students, and our study confirmed the similar effects of this tutoring style in scientific research (30). This superior performance might be attributed to the higher levels of academic effort and generally positive emotions exhibited by students under authoritative mentorship, who engage more robustly in academic activities on emotional, cognitive, and behavioral levels (31, 32). This primarily includes the enhancement of self-efficacy and motivation, which is positively associated with scientific identity and can be linked to the retention in scientific majors (33). While students of permissive and authoritative teachers experience the same level of positive emotions, the lower level of demand of permissive tutors leads to a decreased academic effort among students (34). Conversely, the authoritarian style, often perceived as excessively strict, may undermine students’ motivation and autonomy, consequently resulting in sub-optimal teaching effectiveness (30).

The results indicated that the students of middle-career tutors performed better in terms of academic results compared to those of tutors with early careers. Tutors in the relatively early stages of their careers may have limited expertise and educational skills in the scientific domain (35). Consequently, undergraduates may not receive sufficient guidance or domain-specific knowledge to maximize their scientific and learning outcomes. However, although students could benefit from the higher-level research skills and abilities of late-career tutors, they did not have a better academic performance than students of mid-career teachers. This could be senior tutors have to undertake heavier tasks in clinical work, scientific research, administration, and teaching, etc., making it difficult to provide timely and effective guidance to help students solve problems (36). Similarly, research has found that more experienced teachers have a poorer interest in mentoring student research, which could also be a reason for their students’ poorer academic performance (37). Therefore, tutors with middle-career experience, who possess both experience and energy, may be a better choice for tutoring students.

In summary, this study establishes a meaningful connection between the key design elements of UDBRE and both short-term and long-term student outcomes, providing empirical support for the potential of UDBRE to foster student growth within a model-based framework. However, it is important to acknowledge several limitations inherent to this study. First, the research was conducted exclusively within a single medical school in China, which may limit the generalizability of our findings. Thus, further validation in a larger and more diverse population is essential to strengthen the external validity of our conclusions. Second, this study adopted a cross-sectional design, which provides insights into the current impact of UDBRE. To comprehensively assess the enduring effects, a longitudinal cohort study tracking students over time would be a valuable addition to the research. Finally, it is important to recognize that students’ motivation plays a critical role in influencing the outcomes and quality of UDBRE, as the selection process involves mutual agreement between tutors and students. While the full participation and uniform academic requirements of UDBRE can mitigate self-selection bias to some extent, future research should delve into variables related to academic motivation and preparedness. This would contribute to a more comprehensive understanding of the impact of UDBRE participation itself.

## Conclusion

This study provides insights into the efficacious mentorship in UDBRE on scientific research of medical undergraduates in China. The results demonstrated that UDBRE engagement positively impacts students’ performance in scientific research including their mastery of scientific research abilities and attributions in scientific research projects and academic outputs. Furthermore, high-quality of feedback, the authoritative tutoring style and the tutor with a middle-career experience had a positive predictive effect on students’ academic performance. These findings offer useful suggestions and strategies to enhance the training effectiveness of UDBRE. They may also provide guidance for the future design and implementation of UDBRE programs.

## Data availability statement

The original contributions presented in the study are included in the article/supplementary material, further inquiries can be directed to the corresponding authors.

## Ethics statement

The studies involving human participants were reviewed and approved by the Institutional Ethics Committee of Guangzhou Medical University. Written informed consent to participate in this study was provided by the participants.

## Author contributions

XM: Data curation, Investigation, Writing – original draft. XC: Conceptualization, Investigation, Writing – review & editing. JL: Data curation, Investigation, Writing – original draft. ZW: Writing – review & editing. LG: Funding acquisition, Writing – review & editing, Methodology, Project administration. SL: Writing – review & editing, Conceptualization, Supervision, Validation. TL: Supervision, Writing – review & editing, Funding acquisition.
